# 基于聚丙烯酰胺凝胶电泳结合在线荧光成像的蛋白质特异性定量分析方法

**DOI:** 10.3724/SP.J.1123.2024.12017

**Published:** 2025-09-08

**Authors:** Rui ZOU, Zixian YU, Zehua GUO, Haozheng DAI, Qiang ZHANG, Weiwen LIU, Chengxi CAO

**Affiliations:** 上海交通大学自动化与感知学院，上海 200240; School of Automation and Sensing，Shanghai Jiao Tong University，Shanghai 200240，China

**Keywords:** 凝胶电泳, 在线荧光成像, 特异性检测, 蛋白质定量分析, gel electrophoresis, online fluorescence imaging, specific detection, protein quantitation

## Abstract

蛋白质的特异性检测在生物分析和临床诊断中具有重要意义。现有方法如免疫固定电泳和蛋白质印迹法，需要手工染色和脱色等步骤，操作繁琐，耗时久且定量能力有限。本文结合在线荧光成像技术开发了一种蛋白质免疫聚丙烯酰胺凝胶电泳（PAGE）定量检测方法。该方法首先采用荧光标记抗体结合目标蛋白质，使用甲醛交联免疫复合物后进行电泳和在线成像，最终通过计算凝胶电泳图像中游离抗体的荧光信号强度实现定量分析。整个检测流程可在1.5 h内完成。本文以人转铁蛋白（transferrin，TRF）为模式蛋白进行方法学验证，结果显示该方法的线性范围为5.0~200.0 mg/L，线性相关系数（*R*^2^）为0.993 0，制作标准曲线的标准溶液中荧光信号强度的最大相对标准偏差（RSD）为1.65%，检出限为0.5 mg/L，加标回收率为98.2%~105.0%，表明该检测方法具有良好的精密度、灵敏度和准确度。相较于传统的PAGE方法，该方法利用实时荧光成像技术实现了对目标蛋白质的在线定量检测，具有分辨率高、特异性强、操作简便、分析快速以及重现性好等优点。该方法具有普适性，可应用于其他蛋白质的定量检测，在药物制备和临床诊疗等领域具有重要的应用价值。

## Abstract (en)

Specific protein detection plays a crucial role in biological analysis and clinical diagnostics， serving as an essential tool for disease diagnosis， therapeutic monitoring， and biological research. However， conventional methods such as immunofixation electrophoresis （IFE） and western blotting （WB） suffer from complex workflows， time-consuming operations， and limited quantification capabilities owing to intricate staining and de-staining procedures. In addition， these traditional immunological detection methods require extensive manual handling and specialized expertise， while low levels of automation restrict their applicability to high-throughput or large-scale analysis scenarios. Moreover， the multistep nature of these methods increases the risk of experimental errors and compromises quantification accuracy.Herein， we present a quantitative protein immune polyacrylamide gel electrophoresis （PAGE） detection method that combines immune-recognition principles with online fluorescence imaging technology， thereby offering a rapid and specific approach for quantifying target proteins. The developed method exploits the specificity of fluorescently labeled antibodies and the separation capability of PAGE， with formaldehyde crosslinking used to stabilize antigen-antibody complexes under the denaturing conditions of electrophoresis， thereby ensuring reliable quantification. The entire experimental workflow can be completed within 1.5 h and consists of three main steps. Firstly， the target protein is incubated with fluorescently labeled antibodies at room temperature for 0.5 h to form immune complexes， after which they are crosslinked using formaldehyde. The cross-linked samples are then loaded onto polyacrylamide gels and separated under optimized electrophoresis conditions （120 V， 15 min； 150 V， 15 min； 200 V， 15 min）. Electrophoretic separation is finally monitored in real-time using an online fluorescence imaging system， which enables direct visualization of protein migration and eliminates the need for post-separation processing. Three distinct bands are observed on the precast gel following immune PAGE separation： the immune complexes at the uppermost position， the free fluorescent antibodies in the middle， and other proteins at the bottom. ImageJ software is used to analyze the electrophoresis pattern， and quantification is achieved based on the linear relationship between the fluorescence intensity of the free antibody and the mass concentration of the target protein. We systematically validated the performance of the method using human transferrin （TRF） as the model antigen protein and fluorescein-isothiocyanate-labeled （FITC-labeled） anti-TRF IgG antibody （anti-TRF IgG-FITC） as the detection probe， which involved analyzing three key aspects： the necessity of the formaldehyde-crosslinking step for maintaining immune complex stability， antibody recognition specificity in complex samples， and the linear correlation between the fluorescence signal and the mass concentration of the target protein. The method exhibited excellent analytical performance， with a linear range that extended between 5.0 and 200.0 mg/L and a correlation coefficient （*R*^2^） of 0.993 0. Triplicate measurements of fluorescence intensity at all mass concentration points revealed a maximum relative standard deviation （RSD） of 1.65%. The limit of detection （LOD） reaches 0.5 mg/L， with recoveries between 98.2% and 105.0%. Repeatability experiments revealed maximum intra- and inter-day RSDs of 1.21% and 1.58%， respectively. Specificity testing confirmed that the developed method accurately quantified TRF without interference from other proteins in complex samples. These results highlight the good accuracy， excellent consistency， high sensitivity， and robust specificity of the developed method， thereby confirming its reliability for use in precise protein-quantification applications. Compared to traditional PAGE methods， the immune PAGE method introduced herein provides the ability to selectively quantify specific target proteins online using real-time fluorescence imaging technology. The method exhibits several notable advantages， including high resolution， a simple workflow， strong specificity， rapid analysis， and good reproducibility. The online fluorescence imaging system eliminates the need for complex gel-dismantling， membrane-transfer， fixation， staining， and de-staining steps while effectively preventing protein band broadening， thereby enabling highly sensitive quantitative analyses with superior resolution. The cost-effectiveness of the developed method is achieved through economical fluorescently labeled antibodies and low sample consumption. Moreover， the fundamental principle of the immune PAGE method suggests that this approach is readily adaptable to the quantification of other proteins through the judicious selection of specific fluorescently labeled antibodies. Consequently， the versatility of the developed method makes it a comprehensive analytical platform suitable for pharmaceutical preparations and clinical diagnostics， in which rapid and accurate protein quantification is essential for decision-making processes. Additionally， this method is an ideal choice for high-throughput applications in both academic research and industrial settings owing to the integration of automated analysis and short operation times. The protein immune PAGE method represents a significant advancement in specific protein quantification methodology with great potential as a promising tool that is expected to be widely adopted in various biological analysis scenarios.

蛋白质的精确定量分析^［1，2］^对于阐明生物学机制^［3］^和理解各种疾病的发病机理^［4］^具有重要意义。聚丙烯酰胺凝胶电泳（polyacrylamide gel electrophoresis，PAGE）^［5，6］^是目前使用最广泛的蛋白质检测和纯度分析技术之一，其分离原理是不同蛋白质分子在电场作用以及凝胶分子筛效应下会表现出不同的电泳迁移率^［7］^。然而，传统的PAGE技术缺乏对复杂样品中目标蛋白质特异性识别的能力，也无法对蛋白质浓度准确定量。目前已发展出许多免疫学检测方法用于蛋白质的特异性检测，例如蛋白质印迹法（Western blotting，WB）^［8］^、酶联免疫吸附测定法（enzyme-linked immunosorbent assay，ELISA）^［9］^和免疫固定电泳法（immune fixation electrophoresis，IFE）^［10，11］^等。

WB和ELISA均可使用特异性抗体实现对目标蛋白质的靶向检测。然而，WB存在操作复杂、耗时较长且重复性较差的问题^［12］^，更重要的是，其检测能力局限于定性或半定量水平^［13］^。尽管ELISA和IFE能够实现蛋白质的特异性定量检测，但这两种方法都存在试剂成本昂贵、假阳性率较高的问题^［14，15］^。此外，WB、ELISA和IFE的检测流程均需要依赖经验丰富的操作人员来完成，这种低自动化程度严重限制了以上技术在大规模应用场景中的推广^［11，12，16］^。虽然商业化的毛细管电泳仪已经可以实现自动化的蛋白质定量分析，但其分离方案相对严苛，需要针对不同种类的蛋白质进行特定的电泳条件优化^［17］^。

近几年来，PAGE技术结合在线光学成像系统在蛋白质的分离检测方面取得了显著进展。Xue等^［18，19］^开发了自冷却凝胶电泳芯片用于高效蛋白质分离，并基于该芯片发展了一种在线本征荧光成像（intrinsic fluorescence imaging，IFI）系统用于蛋白质的免染料检测。Yu等^［20］^在Xue等先前工作的基础上，重新设计了在线IFI系统的紫外光场分布并优化了PAGE装置，开发了一种与标准平板凝胶相兼容的PAGE-IFI方法，可实时监测蛋白质的电泳分离进程，并能依据对电泳图像的分析实现蛋白质的准确定量。然而，结合在线光学成像系统的PAGE技术尚未应用于复杂样品中目标蛋白质的特异性定量分析。

本文基于在线荧光成像技术开发了一种蛋白质免疫PAGE方法，可实现复杂样本中目标蛋白质的特异性定量检测。以人转铁蛋白（transferrin，TRF）和荧光素异硫氰酸酯（fluorescein isothiocyanate，FITC）标记的抗人TRF IgG抗体（anti-TRF IgG-FITC）分别作为模式抗原和抗体，对本方法进行了性能验证。结果表明，相较于传统的蛋白质免疫分析方法，本方法无需复杂的凝胶拆卸、转膜、固定、染色和脱色等步骤，不仅简化了操作流程、缩短了检测时间，而且通过使用在线荧光成像技术有效避免了蛋白质条带的展宽，从而实现了高灵敏度和高分辨率的蛋白质检测分析。

## 1 实验部分

### 1.1 仪器与试剂

ML204电子天平（梅特勒托利多，瑞士）；Mixer-4K微型漩涡混合仪（上海生工生物工程股份有限公司）；MK2000-2E干式恒温器（杭州奥盛仪器有限公司）；TMM-5S魔方程序混匀器（宁波拓普森科学仪器有限公司）。使用超纯水系统（18.2 MΩ·cm，默克密理博，美国）生产去离子水。

重组人脱铁型TRF购自北京索莱宝科技有限公司。FITC偶联的山羊抗人TRF IgG（H+L）多克隆抗体（anti-TRF IgG-FITC）购自武汉三鹰生物技术有限公司。马源肌红蛋白（myoglobin，Mb）购自上海源叶生物科技有限公司。牛源细胞色素C（cytochrome C，Cyt-C）购自北京博奥森生物技术有限公司。C-藻蓝蛋白（C-phycocyanin，C-PC）、甘油和溴酚蓝购自上海麦克林生化科技有限公司。三（羟甲基）氨基甲烷（tris（hydroxymethyl）aminomethane，Tris）购自阿拉丁试剂（上海）有限公司。牛血清白蛋白（bovine serum albumin，BSA）、Tris-盐酸（Tris-HCl）缓冲液（0.5 mol/L，pH 6.8）、甘氨酸（glycine，Gly）、十二烷基硫酸钠（sodium dodecyl sulfate，SDS）、甲醛溶液（HCHO，37%~40%）、Tris-Gly-SDS（TGS）缓冲液粉末和彩色预染蛋白质相对分子质量标准品（10~250 kDa）均购自上海阿达玛斯试剂有限公司。磷酸盐缓冲溶液（phosphate buffered saline，PBS）和商用BeyoGel^TM^ Plus预制PAGE凝胶（Tris-Gly，4%~20%梯度，10孔）均购自上海碧云天生物技术有限公司。

### 1.2 样品制备与实验操作

上样缓冲液：在烧杯中依次加入20 mL Tris-HCl缓冲液、40 mL 10%的SDS溶液、20 mL甘油和500 μL 1%的溴酚蓝溶液，用去离子水定容至100 mL。

电泳缓冲液：取TGS缓冲液粉末，用去离子水溶解并定容至1 L，配制完成的电泳缓冲液含有25 mmol/L的Tris、192 mmol/L的甘氨酸以及0.1%的SDS。

免疫复合物：在不同离心管中分别加入不同体积的TRF溶液（5 mg/mL）和1 μL的anti-TRF IgG-FITC溶液（商品给定质量浓度0.6 mg/mL），用PBS定容至100 μL后得到TRF质量浓度梯度为0、5.0、10.0、20.0、50.0、100.0、200.0、500.0 mg/L的免疫复合物样本。将离心管同时置于魔方程序混匀器上，在振动模式6下室温孵育0.5 h，使抗原抗体结合，形成免疫复合物，随后加入甲醛至终浓度为1%，交联已形成的复合物，再加入Tris至终浓度为20 mmol/L，淬灭多余的甲醛分子。最后将免疫复合物样本与上样缓冲液以1∶1的体积比混合，在95 ℃下加热5 min即可用于后续电泳上样。

电泳与荧光成像条件：选择曝光时间3 000 ms和增益15的拍摄参数进行荧光成像，在加样前预先采集凝胶的背景信号。所有样品均独立制备3次，用于3组平行实验测定。将制备好的免疫复合物样本（10 μL/孔）加载至预制PAGE凝胶的样品孔中，采用阶梯电压程序进行电泳分离：初始以120 V恒压电泳15 min，随后升至150 V电泳15 min，最后以200 V恒压电泳15 min。电泳后使用前述相同参数拍摄获得凝胶电泳荧光图像。

图像处理：采用商用图像处理软件ImageJ（NIH，美国）对凝胶电泳荧光图像进行分析，首先减去预先采集的凝胶背景信号，随后使用多边形工具划分出不同样品中游离荧光标记抗体对应的条带区域，最后通过对各区域进行灰度值积分来定量游离抗体的荧光信号强度。

### 1.3 蛋白质免疫PAGE定量检测方法

基于实验室先前的研究工作中开发的在线荧光成像系统^［21］^，本文开发了一种蛋白质免疫PAGE定量检测方法。如图1a所示，首先用荧光标记的抗体（anti-TRF IgG-FITC）特异性结合目标抗原（TRF），形成免疫复合物，再利用甲醛交联复合物以保证其在后续电泳过程中的稳定性^［22］^。

**图1:**
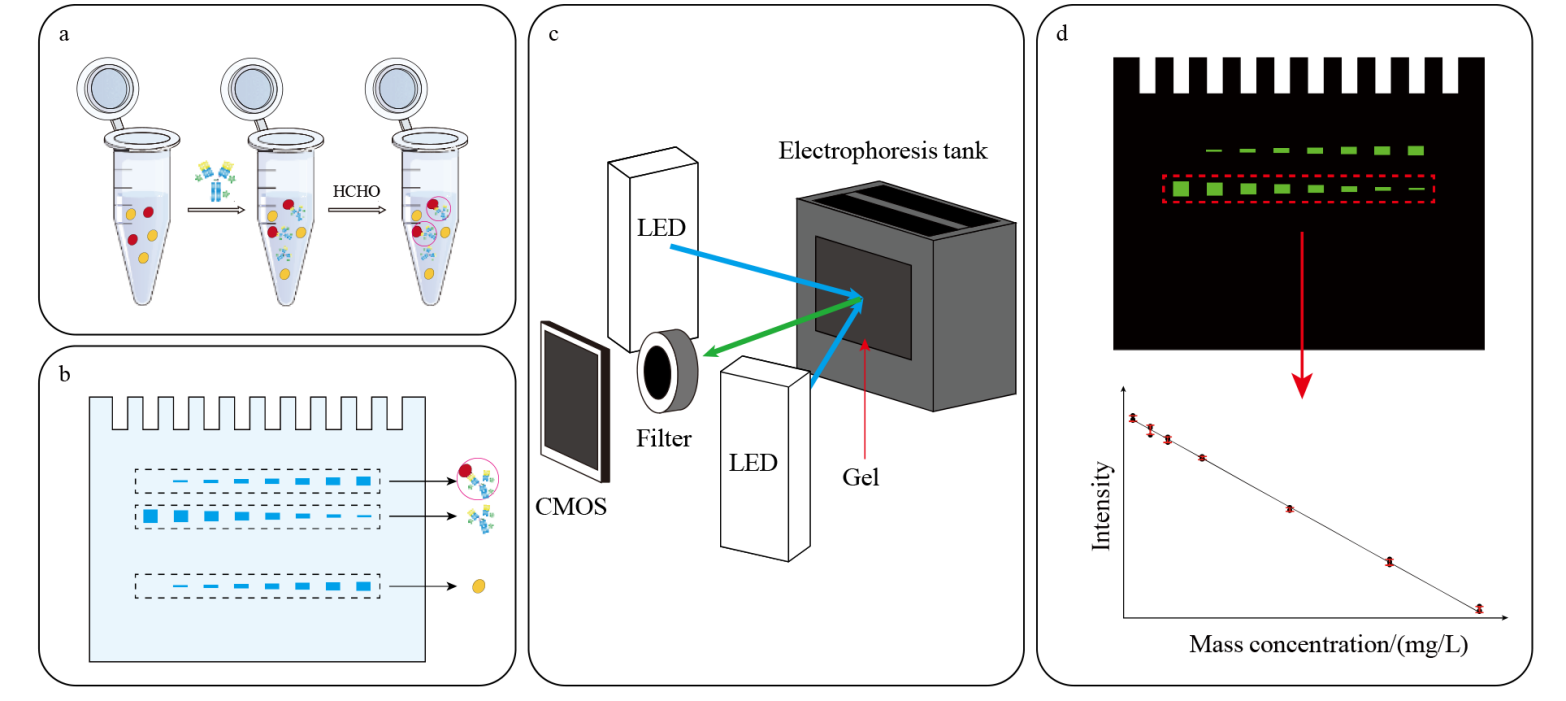
免疫PAGE定量检测方法的原理示意图 a. illustration of immunological binding and formaldehyde crosslinking of immune complex of transferrin （TRF） and fluorescein-isothiocyanate-labeled （FITC-labeled） anti-TRF IgG antibody （anti-TRF IgG-FITC）； b. diagram of protein separation by electrophoresis； c. optical path configuration of the online fluorescence imaging system； d. principle of gel fluorescence imaging and quantitative detection of the immune complex and anti-TRF IgG-FITC.

图1b为免疫PAGE分离不同蛋白质组分的示意图，其中最上方的是免疫复合物条带，中间的是游离的荧光标记抗体条带，最下方的是由杂蛋白形成的条带。在此过程中，每组样品均含有相同浓度的荧光标记抗体，而不同样品中目标蛋白质浓度不同，因此消耗的荧光标记抗体量也不同。目标蛋白质浓度越高，则未结合目标蛋白质的游离荧光标记抗体就越少，反映在中间条带的荧光信号强度则越低。

图1c所示的在线荧光成像系统既可以实现荧光信号的在线捕获，又可以实时采集电泳分离荧光图像。随后通过ImageJ分析凝胶电泳荧光图像，获取各泳道中游离抗体条带的荧光信号强度。将已知浓度的标准样品中目标抗原蛋白质的浓度与其对应的游离抗体荧光信号强度进行线性拟合，建立免疫PAGE定量分析标准曲线，如图1d所示。在实际检测时，将待测样品按照相同的实验条件进行免疫PAGE分离，通过测定游离抗体的荧光信号强度，结合标准曲线即可换算得到样品中目标蛋白质的浓度。

## 2 结果与讨论

### 2.1 甲醛交联的作用

抗原与抗体的反应为非共价结合，其在电泳过程中容易解离，因此需要利用甲醛交联蛋白质技术有效固定抗原-抗体复合物^［22］^。为验证甲醛交联在免疫PAGE中的必要性，进行了对比实验：将质量浓度为0、5.0、10.0、20.0、50.0、100.0、200.0、500.0 mg/L的TRF与1 μL的anti-TRF IgG-FITC（0.6 mg/mL）溶液混合，共同孵育形成免疫复合物样本。然后将样本分成两组，一组使用甲醛交联，另一组不作任何处理。分别将两组样本加载至两块相同的预制胶上，使用在线荧光成像系统对其进行相同的免疫PAGE程序，并使用相同的拍摄参数（曝光时间3 000 ms，增益系数 15）采集荧光图像，结果分别如图2a和图2b所示。

**图2:**
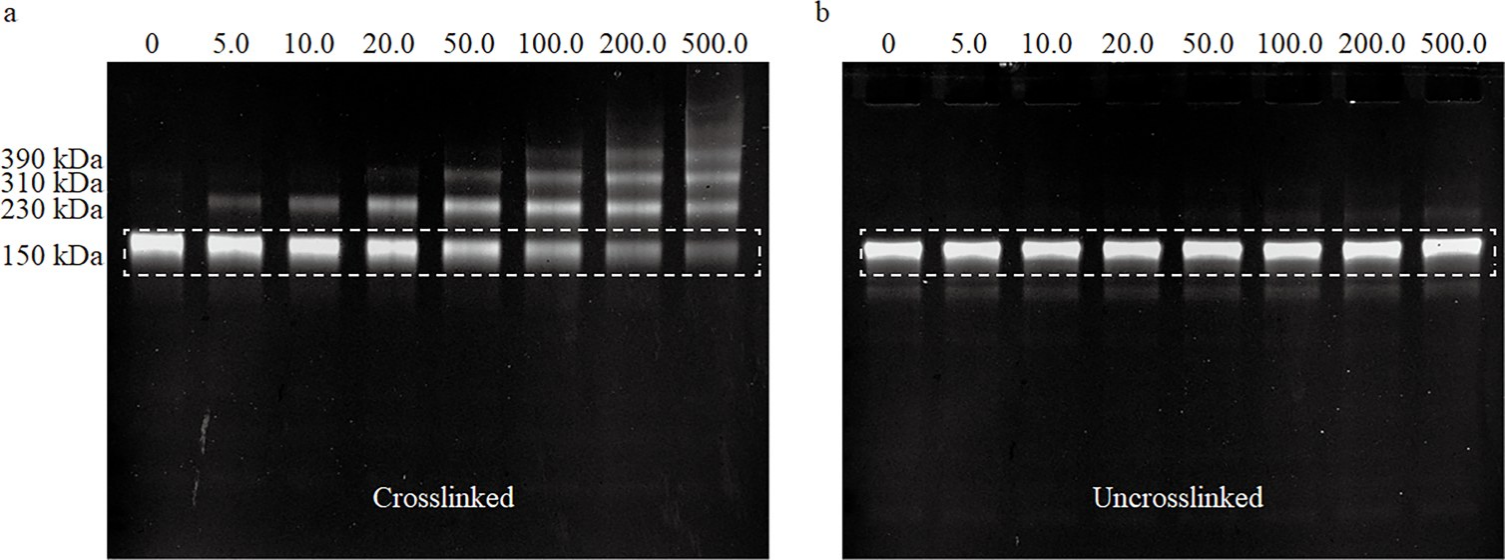
有/无甲醛交联时免疫复合物电泳稳定性的对比 a. fluorescence imaging of immune complexes formed between TRF antigen and anti-TRF IgG-FITC after formaldehyde crosslinking treatment； b. fluorescence imaging of the same immune complexes without formaldehyde crosslinking. The numbers above each lane indicate TRF mass concentrations （mg/L） in the immune complex samples， and protein relative molecular masses are marked on the left.

其中图2a为使用甲醛交联免疫复合物获得的凝胶电泳荧光图像，可以观察到150 kDa处的游离抗体条带的荧光信号强度随着TRF浓度的升高而逐渐减弱。并且在150 kDa的条带上方出现一系列高相对分子质量复合物条带（230、310和390 kDa），可能的原因是anti-TRF IgG-FITC存在多个免疫结合位点，因而形成了多个相对分子质量的免疫复合物。而在不使用甲醛交联处理的图2b中，不同TRF质量浓度的免疫复合物样本在150 kDa处条带的荧光信号强度之间却无明显差异（RSD=3.11%）。两组样本的对比结果表明，在没有甲醛交联的情况下，已经结合的抗原抗体免疫复合物会在PAGE过程中再次解离，从而干扰目标蛋白质的准确定量。因此，通过甲醛交联维持免疫复合物在PAGE过程中的稳定性是实现准确定量检测的关键步骤，这也是本工作建立的蛋白质免疫PAGE定量检测方法中的重要环节。

### 2.2 特异性检测验证

为了验证蛋白质免疫PAGE定量检测方法的特异性，制备了6组样品，分别含有不同的蛋白质组分，每组样品中含有的蛋白质见表1。将6组样品与1 μL的anti-TRF IgG-FITC溶液混合孵育，并设置一个不含抗原蛋白质的空白对照组，最终7个反应体系中anti-TRF IgG-FITC的质量浓度均为6.0 mg/L。随后将7组样品加载至同一块预制胶上，使用在线荧光成像系统对它们同时进行PAGE并拍摄凝胶电泳荧光图像，计算荧光图像中各实验组在150 kDa处的游离抗体条带的信号强度与空白对照组条带的信号强度之差，图3为各实验组与空白对照组的游离抗体荧光信号强度差值条形图。可以看到，仅在含有TRF的实验组样本（样本1和样本6）与空白对照组之间出现明显的荧光信号差值，而其余实验组样本的荧光信号强度与空白对照组差异较小。这些对比实验结果证实了anti-TRF IgG-FITC对TRF具有特异性识别的能力，为本方法实现在复杂样本中对目标蛋白质的特异性定量检测提供了有力的理论依据。

**表1:** 特异性验证实验中各样本的蛋白质组分

Sample	TRF	BSA	C-PC	Mb	Cyt-C	anti-TRF IgG-FITC
1	**+**	**-**	**-**	**-**	**-**	**+**
2	**-**	**+**	**-**	**-**	**-**	**+**
3	**-**	**-**	**+**	**-**	**-**	**+**
4	**-**	**-**	**-**	**+**	**-**	**+**
5	**-**	**-**	**-**	**-**	**+**	**+**
6	**+**	**+**	**+**	**+**	**+**	**+**
Blank	**-**	**-**	**-**	**-**	**-**	**+**

“+” indicates the presence of corresponding protein in the sample. For antigen proteins， “+” represents a mass concentration of 50.0 mg/L； for anti-TRF IgG-FITC， “+” indicates its final mass concentration is 6.0 mg/L. “-” indicates the absence of corresponding protein in the sample.

**图3:**
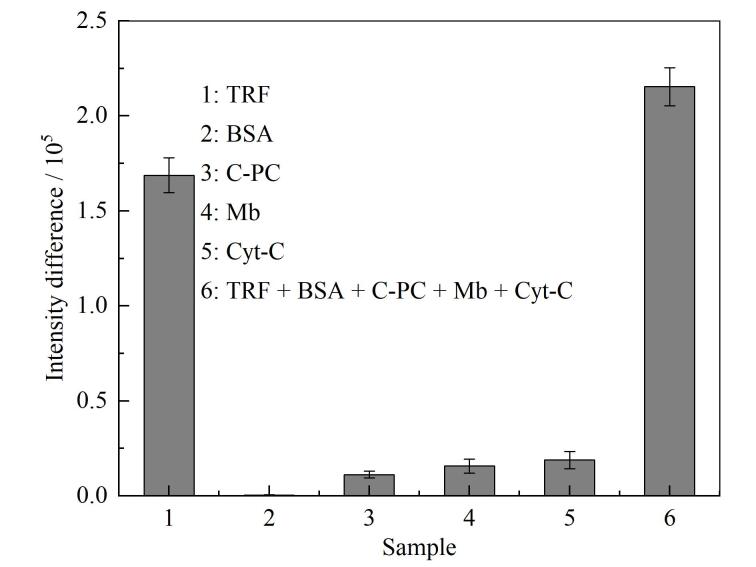
蛋白质免疫PAGE方法特异性验证的荧光信号差值条形图

### 2.3 TRF的定量检测

本文基于游离荧光标记抗体的信号强度进行目标蛋白质的定量检测。在电泳过程中，荧光标记抗体条带的信号强度与溶液中抗体浓度的线性相关性是后续目标蛋白质定量检测的基础。为了验证这一线性关系，将购买的anti-TRF IgG-FITC溶液（0.6 mg/mL）用PBS按不同体积比（1∶10、1∶20、1∶40、1∶50、1∶100、1∶200）分别稀释。使用在线荧光成像系统对其进行电泳和荧光图像采集，并分析电泳图像中条带的荧光信号强度与anti-TRF IgG-FITC溶液浓度的线性关系。如图4a所示，条带的荧光信号强度与溶液中抗体的浓度呈显著线性相关，相关系数（*R* ^2^）为0.994 5，对每个anti-TRF IgG-FITC溶液进行了3次平行实验测定，计算得到各浓度下荧光信号强度的RSD<5.41%（*n*=3），充分证明荧光信号强度与抗体浓度具有线性关系。

**图4:**
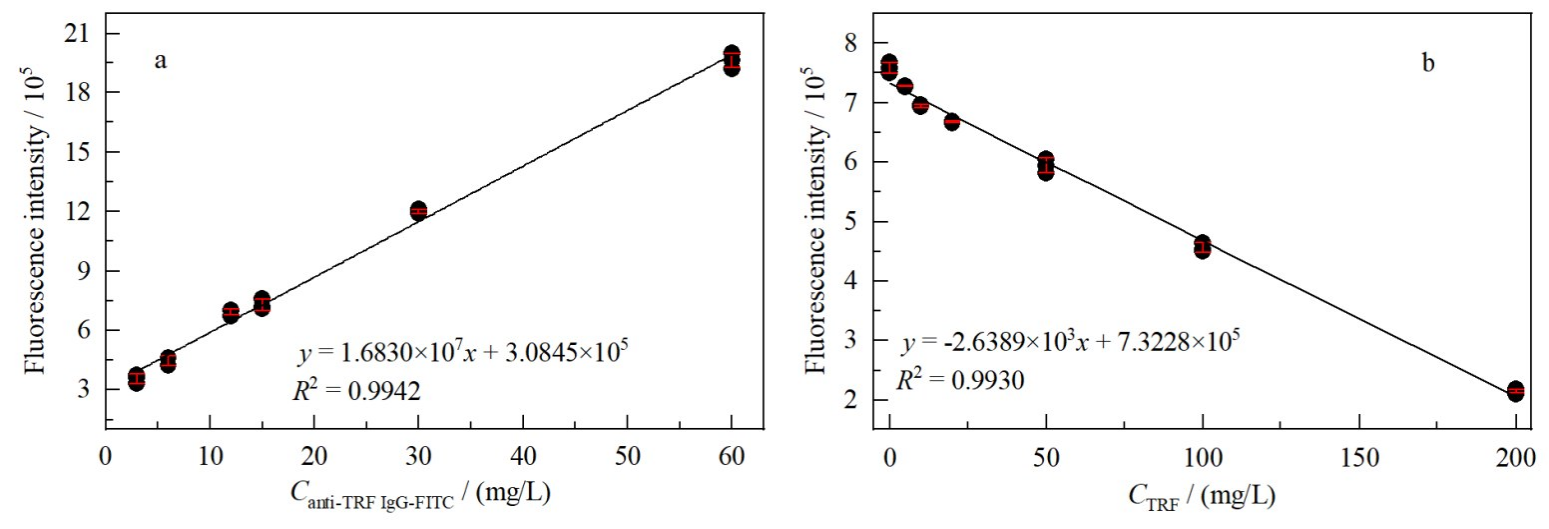
使用TRF和anti-TRF IgG-FITC验证免疫PAGE方法性能的实验结果（*n*=3） a. linear correlation between band grayscale intensity and anti-TRF IgG-FITC mass concentration； b. calibration curve of fluorescence intensity versus TRF mass concentration.

TRF是人体内主要的铁离子转运蛋白，在中枢神经系统铁稳态调控中发挥着关键作用^［23］^，而血清中转铁蛋白水平的异常与多种神经退行性疾病的产生密切相关^［24，25］^，因此快速准确的转铁蛋白定量检测在疾病的早期诊断^［26］^、治疗监测^［27］^和预后评估^［28］^中具有重要意义。本文选取TRF作为目标抗原蛋白质考察免疫PAGE的方法性能，为了建立TRF浓度定量的标准曲线，制备了TRF质量浓度为5.0、10.0、20.0、50.0、100.0、200.0 mg/L的免疫复合物样本。使用在线荧光成像系统进行蛋白质电泳分离和荧光图像采集，并分析凝胶电泳荧光图像中各样品中游离的anti-TRF IgG-FITC在150 kDa处所产生的条带的荧光信号强度，将样品中TRF的浓度与荧光信号强度一一对应，得到如图4b所示的TRF浓度定量标准曲线。其中每个TRF浓度的荧光信号强度均由3次平行实验测定，所有浓度的荧光信号强度最大RSD 为 1.65%（*n*=3），线性拟合的相关系数*R* ^2^为0.993 0，标准曲线的斜率绝对值为2.638 9×10^3^。为了确定方法的检出限（LOD），制备不含TRF的空白对照组样品并进行相同的免疫PAGE程序，分析所得条带的荧光信号强度的RSD，计算得到本方法的LOD为0.5 mg/L。继续对本方法进行重复性评估，结果显示：日内RSD（*n*=3，间隔2 h）最大值为1.21%，日间RSD（*n=*3，间隔48 h）最大值为1.58%，表明本文提出的蛋白质免疫PAGE定量检测方法在TRF浓度检测上具有良好的可靠性。

为了进一步验证本方法在复杂样本中定量检测TRF的能力，制备了同时含有TRF、BSA、C-PC、Mb和Cyt-C的多组分蛋白质混合样品，其中TRF的浓度梯度设置为200.0、100.0和50.0 mg/L。使用anti-TRF IgG-FITC对混合样本中的TRF进行特异性捕获，利用在线荧光成像系统运行免疫PAGE程序并采集凝胶电泳图像。结合图4b中所示的TRF浓度定量标准曲线，计算得到3个浓度梯度下样本的TRF加标回收率分别为98.2%、104.8%和105.0%。对每个TRF加标水平进行了3次平行实验测定，分别计算各加标水平下回收率的RSD，结果均小于3.30%，这些数据充分说明了本方法在复杂溶液环境中进行TRF定量分析的准确度。

### 2.4 分析方法优势及应用前景

以上实验结果表明本方法具有以下优点。第一，操作简便高效，无需凝胶拆卸、转膜、固定、染色和脱色等步骤，降低了实验操作引入的误差，整个工作流程可在1.5 h内完成。第二，分析性能优异，以TRF为模型蛋白，基于游离anti-TRF IgG-FITC抗体的荧光信号强度进行定量，本方法LOD达0.5 mg/L，线性范围为5.0~200.0 mg/L（*R* ^2^=0.993 0），建立标准曲线时荧光信号强度的最大RSD为1.65%（*n=*3），加标回收率为95%~105%。第三，具备实时监测能力，通过在线荧光成像系统可实时观察电泳分离全过程，有效防止了蛋白质条带展宽，确保了定量分析的准确性和高分辨率。第四，通用性潜力和成本效益好，本方法有望通过更换特异性抗体用于其他目标蛋白质的定量分析，同时采用经济型荧光标记抗体降低了成本。

将本方法与考马斯亮蓝（Coomassie Brilliant Blue，CBB）凝胶染色法和两种常规的基于平板凝胶电泳的蛋白质免疫学检测方法WB和IFE进行对比分析。如表2所示，这几种方法在特异性识别能力、定量能力、在线成像能力和免染色能力4个关键性能指标上存在显著差异。本文开发的蛋白质免疫PAGE方法通过荧光标记抗体的特异性识别，结合在线荧光成像技术，实现了对目标蛋白质的特异性定量检测，具有良好的定量能力。相比之下，CBB染色法无需抗体孵育，操作简便，但也因此缺乏特异性识别能力，只能对总蛋白进行染色显示和半定量^［29］^，同时作为一种终点检测方式，它无法实现电泳过程的实时监测^［30］^。IFE虽然在多发性骨髓瘤诊断中具有重要意义^［31］^，通过使用特异性抗体（如抗IgG、抗IgA、抗IgM以及抗κ、抗λ轻链抗体）可定量检测不同类型的单克隆免疫球蛋白^［32］^，但它存在一定的假阳性风险^［15］^，且缺乏在线成像功能，需要后续的染色步骤来显示蛋白质条带^［11］^。WB具有较高的灵敏度和特异性，在检测低丰度样本方面表现突出^［33，34］^，但由于需要复杂的转膜和多次抗体孵育、洗脱等步骤，其定量能力和重复性受限^［12，13］^。综上，本方法在保持特异性识别的同时，还具备定量准确、实时监测和操作简便等优势，为蛋白质的特异性定量分析提供了新的技术方案。

**表2:** 蛋白质免疫PAGE定量检测方法与其他免疫学检测方法的对比

Method	Specificity	Quantification	Online Imaging	Staining Free	Antibody^*^	Refs.
CBB	no	no	no	no	/	［^29^，^30^］
IFE	yes	yes	no	no	1^st^ Ab	［^11^，^15^，^31^，^32^］
WB	yes	no	yes	yes	1^st^ & 2^nd^ Ab	［^12^，^13^，^33^，^34^］
Our work	yes	yes	yes	yes	1^st^ Ab-FITC	/

* Antibody （Ab）： “/” represents no antibody needed； “1^st^ Ab” refers to the primary antibody； “2^nd^ Ab” refers to the secondary antibody； “1^st ^Ab-FITC” indicates an FITC-labeled primary antibody.

近年来，基于荧光免疫的定量分析方法在多种生物标志物的检测领域得到广泛应用。通过选择不同靶向的特异性抗体，该类方法已在临床医学诊断^［35］^和食品安全控制^［36］^等领域实现了对多种化合物的灵敏检测，这种通过更换抗体实现不同靶标检测的应用在传统IFE技术中亦得到验证^［32］^。同时，有研究人员通过使用具有不同波段的荧光染料标记不同抗体，实现了对多靶标的同步检测^［37］^。这些研究成果充分预示了本文所述的特异性荧光定量分析方法具有良好的通用性和应用前景。

## 3 结论

本文基于在线荧光成像系统开发了一种蛋白质免疫PAGE定量检测方法，并使用该方法成功实现了对模式蛋白TRF的特异性定量检测。该方法使用荧光标记抗体特异性识别目标蛋白质，再利用在线荧光成像技术实时监测电泳分离过程，最后将采集的凝胶电泳图像用于蛋白质定量分析。以TRF为模式蛋白的系统验证实验结果表明，本方法具备检测范围宽、线性度好、快速简便、特异性强且全程可视化等优点。通过更换不同的荧光标记抗体，该方法可在未来拓展应用于其他目标蛋白质的检测，是一种具有通用性的蛋白质特异性定量分析方法，在疾病诊疗和药物制备等领域具有重要的应用价值和广阔的发展前景。
